# NOS2 and COX2 impact the spatial landscape of CD8^+^ T cells in ER-breast cancer, providing novel mechanistic insight that drives tumor progression and poor survival

**DOI:** 10.1016/j.redox.2026.104189

**Published:** 2026-04-27

**Authors:** Lisa A. Ridnour, Robert YS. Cheng, William F. Heinz, Leandro L. Coutinho, Erika M. Palmieri, M Cristina Rangel, Stephen K. Anderson, Daniel W. McVicar, Stephen M. Hewitt, Stephen TC. Wong, Xiaoxian Li, Stefan Ambs, Timothy R. Billiar, Sharon A. Glynn, Jenny C. Chang, Stephen J. Lockett, David A. Wink

**Affiliations:** a Cancer Innovation Laboratory, Center for Cancer Research, National Cancer Institute, National Institutes of Health, Frederick, MD, USA; b Optical Microscopy and Analysis Laboratory, Frederick National Laboratory for Cancer Research, Leidos Biomedical Research Inc. for the National Cancer Institute, Frederick, MD, USA; c Faculdade de Medicina da Universidade de São Paulo and Comprehensive Center for Precision Oncology, Universidade de São Paulo, São Paulo, SP, Brazil; d Basic Science Program, Frederick National Laboratory for Cancer Research, Frederick, MD, USA; e Laboratory of Pathology CCR, NCI, NIH, Bethesda, MD, USA; f Houston Methodist Neal Cancer Center, Weill Cornell Medical College, Houston Methodist Hospital, Houston, TX, USA; g Department of Pathology and Laboratory Medicine, East Carolina University, Greenville, NC, USA; h Laboratory of Human Carcinogenesis, CCR, NCI, NIH, Bethesda, MD, USA; i Department of Surgery, University of Pittsburgh Medical Center, Pittsburgh, PA, USA; j Discipline of Pathology, Lambe Institute for Translational Research, School of Medicine, University of Galway, Galway, Ireland

**Keywords:** NOS2, COX2, Immunosuppression, Spatial, Breast cancer

## Abstract

Nitric oxide synthase 2 (NOS2) and cyclooxygenase 2 (COX2) tumor expression present significant obstacles for effective treatment of aggressive tumors including ER-negative breast cancer. Spatial analysis of NOS2, COX2 and CD8 expression in patient tumors has identified mechanisms of treatment inhibition that were further elucidated using the 4T1 mouse model of triple negative breast cancer and live cell culture studies. NOS2 and COX2 activate each other via a feed-forward paracrine mechanism. While NOS2 promotes cancer stemness and the formation of metastatic niches, COX2 mediates CD8^+^ T cell suppression. Quantitative spatial analysis has revealed NOS2/COX2 roles during the temporal progression from immune hot in low COX2 expressing tumors to three immune cold stages in the tumor microenvironment of high COX2 expressing tumors. Type 1: immune cold with stroma-restricted CD8^+^ T cell secretion of interferon gamma, which activates COX2 expression at the tumor margin as well as NOS2 expression at the tumor periphery. Type 2: developing immune desserts lack stroma-restricted CD8^+^ T cells with tumor NOS2 and COX2 restricted to the tumor periphery. Type 3: mature immune desserts exhibit abated NOS2, COX2 and CD8^+^ T cells, and induction of B7H4 and cancer-associated fibroblasts driven by significant tumor hypoxia and necrosis. These three types of immune desserts can coexist with each other and with immune hot regions in the same tumor. The coordinated interplay of NOS2 and COX2 indicates that targeting both these enzymes provides an effective treatment strategy that is supported by ongoing clinical trials demonstrating improved clinical outcomes in patients who have otherwise exhausted treatment options.

## 1 Introduction

Estrogen receptor-negative (ER-) breast cancers, particularly triple-negative breast cancer (TNBC), are considered "immunologically hot" due to high tumor mutational burden (TMB), significant immune cell infiltration, as well as high program death-ligand 1 (PD-L1) and indoleamine 2,3-dioxygenase (IDO) expression when compared to hormone receptor-positive subtypes. Despite this, they pose unique immunological challenges to adaptive immunity because of intense immunosuppression and rapid evolution to evade immune surveillance [1]. The tumor microenvironment tends to exhibit less HLA expression leading to reduced antigen levels and high myeloid-derived suppressor cells (MDSC) that abate T cell activation. This prompted Gruosso et al. to examine the immune microenvironment of these tumors where they identified predictive, spatially distinct tumor immune microenvironments based upon CD8^+^ T cell tumor infiltration [1]. Tumor NOS2 and COX2 have recently been identified as key factors that influence the spatial architecture of tumor immune microenvironments in ER- and TNBC and contribute to the disease progression of these and other aggressive tumors [[2], [3], [4], [5], [6], [7], [8], [9], [10]]. When expressed at high levels, NOS2 and COX2 induce numerous pro-oncogenic pathways leading to increased tumor survival and metastasis [[11], [12], [13], [14], [15], [16], [17]]. In addition to oncogene activation, feed-forward NOS2/COX2 signaling modulates the tumor immune landscape leading to the development of immunosuppressive and drug-resistant niches with increased cancer stemness and metastatic potential [[18], [19], [20]]. Recent studies have extensively examined how NOS2/COX2 relationships influence the spatial architecture of adaptive immunity with respect to therapeutic efficacy and ER- and TNBC patient survival [[2], [3], [4], [5], [6], [7]]. Herein, the impact of these tumor NOS2 and COX2 relationships on adaptive immunity will be discussed, along with their implications for diagnosis, treatment and survival. We propose that spatial analysis of immune mediators could facilitate the development of novel diagnostics and treatments for improved TNBC and ER-breast cancer survival, with implications for other aggressive cancers as well. Importantly, a recent phase 1/2 clinical trial combining taxane with the NOS inhibitor LNMMA and aspirin improved survival in chemoresistant TNBC patients. Therefore, clinically available NOS/COX inhibitors provide inexpensive alternatives for patients with exhausted treatment options.

### 1.1 CD8^+^ T cell tumor infiltration improves patient survival

**Fig. 1:**
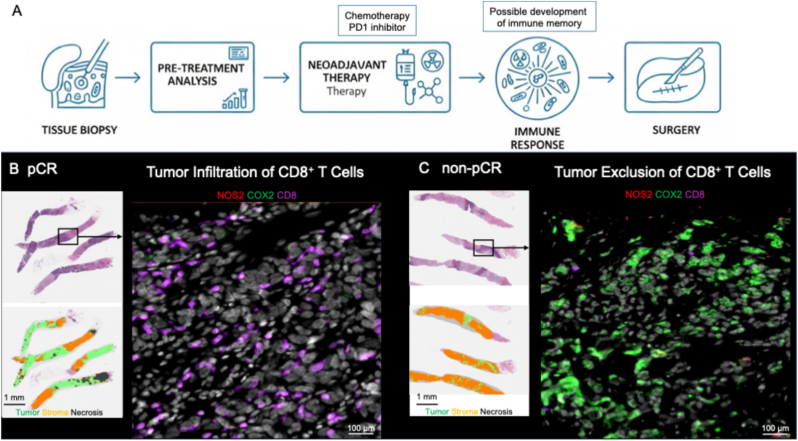
**Comparison of CD8^+^ T cell tumor infiltration associated with pCR vs non-pCR groups in the Keynote 522 clinical trial examining the impact of chemotherapy combined with a PD-1 inhibitor.** A) Schematic representation of TNBC patient analysis and treatment. Keynote 522 clinical trial (approved by the Institutional Review Board of Emory University for human studies previously described [3]) provided research needle biopsies that underwent OPAL staining for NOS2 (red), COX2 (green), and CD8 (purple) for comparison of their expression profiles and spatial localization in tumors from patients achieving B) pCR vs C) non-pCR following treatment. Overlayed H&E-stained serial images annotated for viable tumor (green), stroma (orange), and necrosis (black) were used for the spatial localization of tumor NOS2/COX2 and CD8 expression.

The degree of CD8^+^ T cell infiltration into the tumor core defines hot (high abundance) versus cold (low abundance) tumors, which is a key spatial descriptive of the tumor immune landscape [[21], [22], [23], [24], [25]]. In hot tumors, the accumulation of cytolytic CD8^+^ T cells (T_eff_) in the tumor parenchyma leads to tumor lysis through the release of granzyme B and perforin, which augments antigen presentation and immune memory during tumor eradication [25,26]. Importantly, these inflamed tumors exhibit elevated genomic instability and antigenicity as well as increased proinflammatory cytokines and interferon response mechanisms [2,27]. In contrast, immune desert tumors show abated CD8^+^ T cell penetration, defective antigen presentation, and limited interferon response mechanisms as well as augmented immunosuppressive cells [[27], [28], [29], [30]]. Immune excluded tumors exhibit stroma restricted CD8^+^ T cells that surround the tumor but do not infiltrate into the tumor nest, which suggests impaired activation and/or trafficking [2,29,31]. Thus, effective cytolytic CD8^+^ T cell function is a critical determinant for improved therapeutic efficacy and patient survival and should be exploited for the development of novel immune therapies [2]. Multiple studies indicate that treatment efficacy is contingent upon a proinflammatory immune system [[32], [33], [34]]. In contrast, immune suppression contributes to drug resistance and diminishes treatment efficacy [[35], [36], [37]]. These factors suggest that correct immune polarization may be equally if not more significant than tumor-associated drug resistance pathways [38,39], in part, through drug-induced tumor cell lysis, antigen presentation, and augmented adaptive immunity. The relationship between tumor NOS2/COX2 and CD8^+^ T cell infiltration was spatially explored in tissue biopsies as described in the summary shown in Fig. 1A, where pathological complete response (pCR) vs non-pCR was compared for NOS2/COX2 tumor expression and CD8^+^ T cell infiltration in needle biopsies obtained prior to neoadjuvant chemotherapy and subsequent surgical resection. Fig. 1B shows a favorable onco-immune profile characterized by elevated CD8^+^ T cell tumor infiltration, augmented lymphoid aggregates and tertiary lymphoid structures (TLS) associated with pCR in a low NOS2/COX2 expressing tumor [3]. Importantly, elevated lymphoid structures in the extra tumoral space enhances immune memory development, thereby improving the capacity to eliminate tumors and distal metastatic lesions. This favorable immune profile is abated in non-pCR, high NOS2/COX2 expressing tumors, where CD8^+^ T cells are stroma-restricted with limited tumor infiltration (Fig. 1C).

## 2 NOS2 and COX2 are predictors of poor outcome in ER-breast cancer

**Fig. 2:**
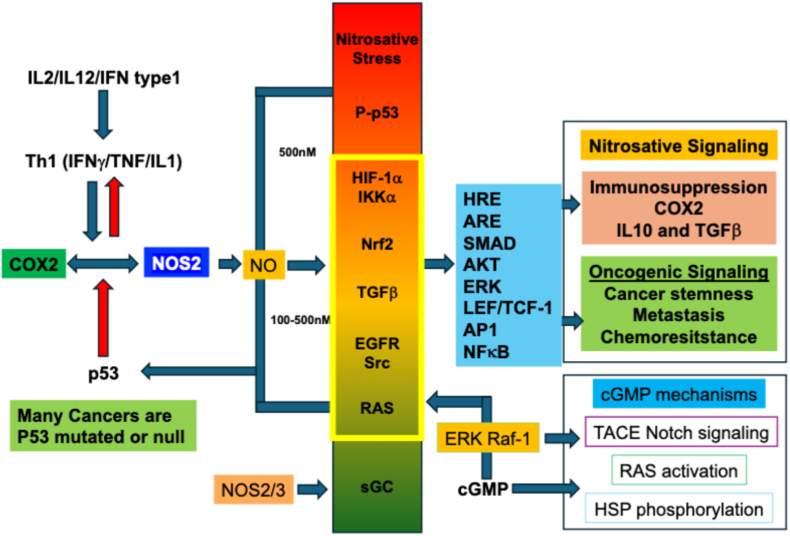
**NO concentration-dependent activation of oncogenic signaling pathways**. Higher NO concentrations that mediated nitrosation signaling (100-500 nM) activate key oncogene signaling. Low NO concentration cGMP-dependent activation of TACE or RAF-1 signaling. WT p53 activation downregulates NOS2 and COX2 expression.

Univariate Cox regression analyses have demonstrated the independent predictive power of tumor NOS2 and COX2 expression in ER-breast cancer with 5-year hazard ratios (HR) of 14.88 (95% CI 2.02-109.5, p = 0.008) and 3.41 (95% CI 1.62-7.17, p = 0.001), respectively [18]. Similarly, multivariate Cox regression confirmed NOS2 (HR = 8.91, 95% CI 1.17-67.6, p = 0.034) and COX2 (HR = 3.03, 95% CI 1.32-6.98, p = 0.009) as independent predictors despite the reduced strength of association of tumor NOS2. These results suggested a potential influence of COX2 on NOS2-related outcome, which was supported by a statistically significant (*p* < 0.0001) interaction between tumor NOS2 and COX2 relative to patient survival. Importantly, stratifying tumor NOS2 and COX2 together increased the HR to 21 (95% CI 2.78-161.9, p = 0.003) [18]. These findings can be explained in part by NOS2/COX2 feed-forward signaling, where NO released from cells with functioning NOS2 induces COX2 in neighboring cells to release PGE2, which induces NOS2 in surrounding cells for increased NO production [18]. Univariate analyses of tumor NOS2 expression have also been correlated with poor outcomes in other aggressive tumors including triple-negative breast cancer, hepatocellular carcinoma associated with HCV, gastric cancer, glioblastoma, stage II colorectal cancer, and stage III melanoma [40]. Disease progression mechanisms have been linked with augmented cancer stem cell markers in chemoresistant and metaplastic cancer, where a significant correlation with NOS2 was identified [4,[41], [42], [43], [44]]. These observations are supported by the induction of chemoresistant proteins in breast cancer cells exposed to NO donors, which was limited by NOS2 inhibitors, [6,7,45,46]. In ER-breast cancer, NO activates oncogenic pathways at concentrations ranging from 100 to 500 nM, which exceeds physiological levels [20,40,47,48]. These excessive NO concentrations augment the nitrosation and activation of targets including EGFR, RAS, SRC, TGFβ, β-catenin, and Nrf2 (Fig. 2) [49]. Furthermore, nitrosylation of non-heme iron sites, such as in prolyl hydroxylase, which regulates proteins including HIF1α, IKKα, as well as histone and DNA demethylases, significantly contributes to the disease progression of cancer [20,40,50]. COX2-associated mechanisms induced by NOS2-derived NO include NFκB and IL-6 expression, which promote cancer stemness [[51], [52], [53], [54]]. At lower cGMP-dependent NO concentrations, the activation of TACE and elevated NOTCH signaling promote HCC disease progression [55,56]. Thus, multiple concentration-dependent NO levels contribute to different oncogenic mechanisms that lead to poor clinical outcomes. Importantly, the NOS2/COX2 axis not only enhances oncogenic signaling but also mediates various immune suppressive mechanisms including increased IL10 and TGFβ that promote immune suppression within the tumor microenvironment [48]. In summary, there are several pro-tumorigenic mechanisms that involve varying levels of NO as well as PGE2 released by COX2 activation, which could in part explain the significant interaction between tumor NOS2 and COX2, and the influence of COX2 on NOS2-related clinical outcomes [18].

## 3 NOS2 and COX2 regulation in normal physiology and cancer

**Fig. 3:**
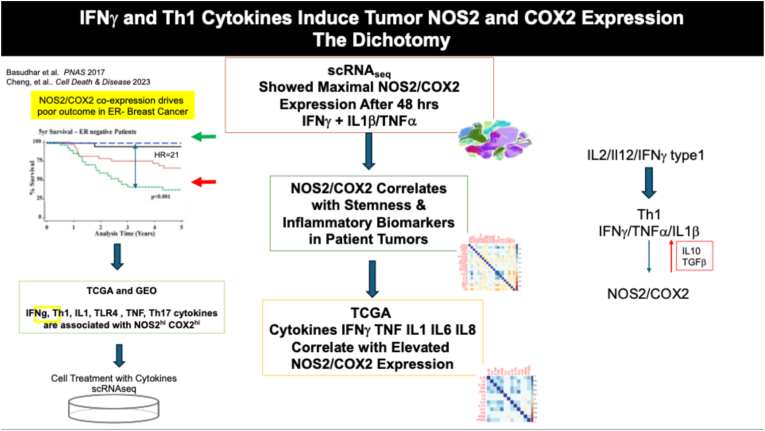
Workflow analysis of ER-tumor microenvironment showing cytokine induction of tumor NOS2 and COX2 expression. Immunohistochemical and transcriptomic analysis of patients revealed Th1 cytokine induction of tumor NOS2 and COX2 that was confirmed by single-cell RNAseq analysis of cytokine treated MB-231 breast cancer cells.

**Cytokine induction of NOS2 and COX2.** Early examination of human hepatocytes showed significant cytokine induced NOS leading to the generation of nitrite and nitrate byproducts [57]. The enzymatic functions of murine and human NOS2 are largely similar, with notable differences in their promoter regions [58]. These observations predict distinct, context-dependent NOS2 expression mechanisms in murine and human systems, which have been confirmed in cell culture experiments and epidemiological studies [59]. In a breast cancer model, the cellular induction of NOS2 and COX2 by cytokine components of the tumor microenvironment was explored in RNAseq data from cytokine stimulated MB-231 breast cancer cells and then compared with NOS2/COX2 low vs high patient tumor signatures [4,5] (Fig. 3). Surprisingly, Th1 cytokines including IFNγ, TNFα, and IL1β, as well as the Th17 cytokine IL17 that are generally associated with improved clinical responses induced NOS2 and COX2 expression in tumors and cells grown in culture [4]. Further examination of single cell RNAseq showed a 48hr exposure to IFNγ combined with TNFα or IL1β significantly induced NOS2 and COX2 expression. In contrast, NOS2 and COX2 expression by IFNγ combined with IL17 or LPS was considerably less. Importantly, IFNγ combined with TNFα induced tumor cell migration and invasion, which was limited by COX2 and NOS2 inhibitors [4,60]. These results indicate that tumor-promoting NOS2 and COX2 expression can be induced by cytokines that mediate anti-tumor immune responses. While these results differ from the Nos2 expression patterns in murine macrophages demonstrating early (<6 h) strong induction by LPS [4,59,61], they are similar to the 1980s studies of Hibbs and coworkers who showed that murine tumor cells exhibited maximal Nos2 induction following 48hr stimulation by IFNγ + TNFα that could indicate an immune-induced tumor stress response [62,63].

NOS2 and COX2 play significant roles during inflammation under normal physiologic conditions, serving as intermediaries between Th1 inflammation, tissue restoration, and resolution of inflammation [17,64]. In cancer, COX2 predicts poor survival in many tumor types and its product PGE2 reduces the production of type I/II interferons, interleukin-2, and interleukin-12 that are required for elimination of tumor cells during immune surveillance [2,17]. Interestingly, wild type p53 can induce anti-tumor immune responses that are abated by p53 mutation [65]. Moreover, wild type p53 downregulates NOS2 and COX2, which does not occur in p53 mutant tumor cells [[66], [67], [68]]. In addition, COX2 inactivates p53 through a physical interaction with amino terminal region aa 1-126 [69]. The inability of mutant p53 to inhibit NOS2 and COX2 expression results in elevated NO and PGE2 levels, triggering a series of protumor and immune suppressive mechanisms observed in these aggressive tumors.

Because NOS2 and COX2 can be expressed under normal physiologic and disease conditions, distinguishing between tumor and host cell NOS2/COX2 expression is critical. Immune cells, including macrophages and monocytes, can be inhibited from acquiring M1 phenotype by factors such as IL-10 and TGF-β, which are essential in suppressing the antitumor immune response. However, IL-10 and TGF-β do not exert the same influence on tumor NOS2 and COX2 expression. The NOS2/COX2 expression dynamics observed between 4 and 12 h in macrophages and 48 h in tumor cells, both in mouse and human models, indicates a temporal response. Thus, IFNγ, TNFα, and IL1β induce NOS2 in both macrophages and tumors but with different temporal profiles [4,59,61]. In the first 24 h, increased NOS2 and COX2 in immune cells subsequently leads to the production of IL-10 and TGF-β, suppressing the immune response. In contrast, tumor NOS2 and COX2 expression occurs independently of IL-10/TGF-β, which suggests a role for tumor NOS2 and COX2 in mediating metastasis, immune suppression, proliferation, and cancer stemness (Fig. 3).

**Orthogonal expression of NOS2 and COX2 in Different Cell Types.** Analyzing the spatial distribution of NOS2 and COX2 across tissue is essential for comprehending the tumor immune microenvironment. Cells exposed to IFNγ/Th1 cytokines induce NOS2 and/or COX2 in many tumor cells, both in mice and to a lesser extent in humans, along with myeloid, endothelial, cancer-associated fibroblasts (CAFs), and adipose tissue [59]. *In vitro* and *in situ* tissue from mice and humans show expression of NOS2 and COX2 in separate cells, i.e. orthogonal expression, with a notable proximity between the two. This expression profile suggests a spatial intercellular paracrine signaling mechanism, with the spatial orientation of these factors impacting distinct properties within the tumor microenvironment.

## 4 Live cell *In vitro* modeling of the tumor microenvironment

In recent years, significant progress has been made in multiplex protein and transcriptomic imaging [[70], [71], [72], [73]]. Multiplex immunofluorescent imaging (MIF) facilitates the analysis of multiple markers alongside spatial transcriptomics. These methods have begun to yield new insights into the distinct characteristics of NOS2 and COX2 cellular neighborhoods [[2], [3], [4], [5], [6], [7]]. Furthermore, mechanisms that have emerged in these small focal environments can be validated in larger GEO and TCGA public databases [[3], [4], [5],^44^,^73^,^74^].

**Fig. 4:**
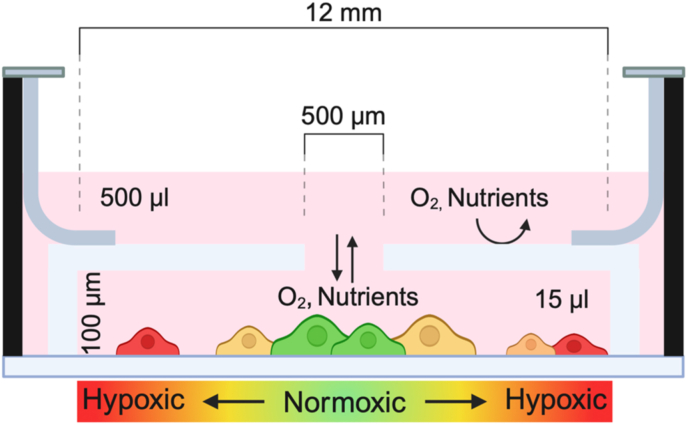
**The REEC design limiting flow to create gradients of O_2_ and nutrients.** Oxygen (and nutrients) decrease from physiological normoxia ([O_2_] > 5%) under the opening to hypoxia ([O_2_] < 1% and anoxia ([O_2_] < 0.1%), typically over radial distances of 1-2 mm from the center of the chamber.

The tumor microenvironment is a complex and heterogeneous entity where specific cellular niches and stromal configurations significantly influence clinical outcomes. It is widely believed that the tumor microenvironment arises from chemical and oxygen gradients that impose polarity on cells and in some cases lead to directed cell migration. To mechanistically examine these conditions in cell culture experiments, we have introduced a live cell *in vitro* mimic, called the Restricted Exchange Environment Chamber (REEC: Fig. 4) [75]. Briefly, the REEC is a multiwell plate insert that partitions a well into two compartments separated by a glass coverslip with a single opening centered on the well. Cells cultured in the lower compartment consume and metabolize soluble molecules, such as oxygen and nutrients, that diffuse through the hole, rapidly generating concentration gradients of those molecules within the lower compartment. For cancer cells cultured at 80% confluence, the oxygen concentration in the REEC decreases from physiological normoxia ([O_2_] > 5%) under the opening to hypoxia ([O_2_] < 1% and anoxia ([O_2_] < 0.1%), typically over radial distances of 1-2 mm from the center of the chamber. Stable gradients result from the equilibrium between diffusion and consumption within 1-2 days, and cells can be cultured in the system at least 30 days. The distribution of cellular phenotypes along the gradients can be readily analyzed by live-cell high resolution confocal fluorescence microscopy, which cannot be done in live 3D spheroid/organoid models. This results in the heterogeneity of the tumor microenvironment that regulates spatially distinct tumor NOS2 and COX2 expression as previously shown by Gilmore et al. [76], where COX2 is expressed within higher O_2_ gradients closer to the opening while NOS2 is expressed more distally at lower O_2_ gradient levels. While chemical and oxygen spatial gradients are non-uniformly ubiquitous in solid tissues, including tumors, these spatial aspects are not considered in most live cell culture systems. Later in this review, we refer to results obtained from using this system that may shed light on the development of immune desert regions.

## 5 The spatial relationship between tumor NOS2/COX2 and CD8^+^ T cells

The accumulation of cytolytic CD8^+^ T cells within the tumor core leads to tumor lysis by CD8^+^ T cells and the recruitment of specific macrophages and dendritic cells. These immune cells are associated with antigen harvesting and processing, which can function as antigen-presenting cells to educate lymphoid cells in the development of both T and B immune cell memory [77,78]. Thus, tumor lysis and antigen presentation are key steps in tumor eradication and cure.

**Fig. 5:**
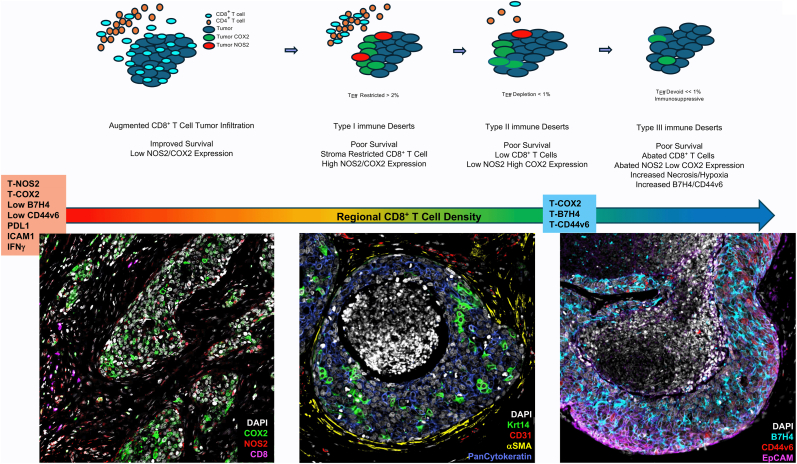
**Distinct CD8^+^ T cell landscape relative to tumor NOS2 and COX2 expression at the tumor edge.** The top schematic shows the development of immune desert regions relative to tumor NOS2 and COX2 expression. Type I immune deserts with stroma restricted CD8^+^T cells as well as NOS2 and COX2 at the tumor margin. Type II immune deserts exhibit fewer CD8^+^T cells with limited NOS2 and reduced COX2 expression. Type III immune deserts have abated CD8^+^ T cells as well as NOS2 tumor expression. Tumor COX2 expression is further reduced in type III immune deserts as B7H4 expression is increased. Type I and III immune desert regions show type I stroma restricted CD8^+^ T cells with large gaps separating them from COX2 expressing tumor. Type III immune deserts show elevated B7H4, EpCAM, and CD44v6 expression. Multiplex fluorescence images are from a retrospective study based upon a historical collection of tumor specimens obtained from patients with breast cancer recruited at the University of Maryland (UMD) Medical Center, the Baltimore Veterans Affairs Medical Center, Union Memorial Hospital, Mercy Medical Center, and the Sinai Hospital in Baltimore between 1993 and 2003. Studies were conducted in accordance with recognized ethical NIH guidelines followed by the Declaration of Helsinki and performed after approval by an institutional review board (IRB) and in accordance with an assurance filed with and approved by the US Department of Health and Human Services under UMD protocol no. 0298229), OHSR no. 2248). This cohort has been published in earlier reports [[2], [3], [4], [5], [6],^8^,^9^,^18^].

Whole tumor imaging has spatially identified tumor immune microenvironments relative to CD8^+^ T cell orientation in TNBC [1]. Four distinct CD8^+^ T cell spatial orientations including fully inflamed immune hot regions, as well as stromal restricted, margin restricted, and immune desert immune cold regions were defined in triple negative breast tumors [1]. Fully inflamed or immune hot tumors exhibited densely distributed CD8^+^ T cells throughout both the tumor stroma and deep into the tumor core. In contrast, stroma restricted tumors displayed high levels of CD8^+^ T cells that were confined in the stroma, with limited or abated penetration into the tumor core. Margin restricted tumors and immune deserts were characterized by fewer than 100 CD8^+^ T cells per mm^2^ [1]. Given that CD8^+^ T cells secrete IFNγ, which induces tumor NOS2 and COX2 expression, we further explored the spatial distribution of CD8^+^ T cells in high vs low NOS2/COX2 expressing ER- and TNBC tumors (Fig. 5) [4,59,79]. This analysis identified stroma restricted CD8^+^ T cells near IFNγ as well as NOS2 and COX2 expressed at the tumor edge, which suggests that CD8^+^ T cells are a plausible source of IFNγ for NOS2 and COX2 induction in tumors [4]. These observations were further explored using the unbiased Spatial Uniform Manifold Approximation and Projection for Dimension Reduction (S-UMAP) described by Giraldo and colleagues [80]. S-UMAP analyzed NOS2, COX2, and CD8 single cell signal intensities in neighboring cells within 25 μm increments over 200 μm ranges to develop phenotype density census neighborhood profiles [5]. This unbiased approach revealed neighborhoods comprised of the CD8^+^NOS2^+^COX2^+^ phenotype in regions with stroma restricted CD8^+^ T cells in deceased patient tumors [5]. This observation supports a role of stroma restricted CD8^+^ T cells as a source of IFNγ in NOS2/COX2 high tumors. In addition, CD8^−^NOS2^−^COX2^+^ neighborhoods were found in immune desert regions, and CD8^−^NOS2^+^COX2^+^ neighborhoods were localized in metastatic niches in NOS2/COX2 high tumors from deceased patients [5]. In contrast, CD8^+^NOS2^−^COX2^-^ neighborhoods defined fully inflamed, immune hot tumors with augmented CD8^+^ T cell tumor penetration that were prevalent in NOS2/COX2 low tumors from Surviving patients [5]. Mechanistically, PGE2 released from high COX2-expressing tumors can impair the interaction of conventional dendritic cells (cDC1) with CD8^+^ T cells to limit recruitment of CD8^+^ T cells to the tumor site [31,81]. PGE2 promotes cDC1 dysfunction by downregulating the transcription factor IRF8 required for antitumor immune response as well as chemokines and cytokines required for the directional migration of cytolytic CD8^+^ T cells into the tumor core [31]. Affected chemokines and cytokines were examined in an RNAseq dataset from 4T1 tumor bearing mice, which showed augmented IRF8, CLEC9a, CXCL9, CXCL10, CXCL11, and IL27 in mice treated with the COX2 inhibitor indomethacin [5]. Moreover, NOS2/CD8 (HR 5.67, 95% CI 1.80-17.84, p = 0.003) and COX2/CD8 (3.34, 95% CI 1.07-10.41, p = 0.038) ratios predicted poor 5-yr survival in the larger Gene Expression Omnibus transcriptomic dataset [[3], [4], [5]]. Together, these results provide evidence supporting the significant interaction between tumor NOS2 and COX2, and the influence of COX2 on NOS2-related clinical outcomes described earlier [18].

## 6 Elevated tumor NOS2/COX2 expression promotes immune desert phenotypes

The Immunoscore is a standardized and worldwide accepted scoring system for colorectal cancer that quantifies CD3^+^ and CD8^+^ T cell density in the tumor core and invasive margin of colorectal tumors [82,83]. Validation studies have shown that Immunoscore outperforms other prognostic indicators including TN stage and lymphovascular invasion, microsatellite instability status, and tumor differentiation [82,83]. While promising, further stratification of stromal tumor-infiltrating lymphocytes in TNBC patients (NCT02685059) revealed no statistical significance as a predictor of disease-free survival and pCR [84]. These findings warrant a more complete description of the tumor immune microenvironment in TNBC and other aggressive breast tumors.

Whole tumor imaging has revealed key differences in tumor NOS2/COX2 expression in immune cold tumors [3]. As stated above, fully inflamed immune hot tumors were identified in low NOS2/COX2 expressing ER- and TNBC breast tumors [6]. Mechanistic exploration in the 4T1 mouse model revealed increased antigen presentation, CD8^+^ T cell tumor infiltration, proinflammatory cytokines and interferon responses, as well as augmented mature B cell populations [2,3,6]. In contrast to these proinflammatory anti-tumor responses, NOS2/COX2 high tumors exhibited three levels of CD8^+^ T cell exclusion, including type I inflamed regions, type II developing immune deserts, and type III mature immune deserts, which may all coexist in individual tumors (Fig. 5) [3]. Type I inflamed regions exhibited stroma restricted CD8^+^ T cells, elevated NOS2^+^ tumor cells at the tumor margins and high COX2 expression at the margin and deeper into the tumor bed. Type II developing immune deserts were NOS2^-^ at the tumor margin with high COX2 expression. Type III mature immune deserts also exhibited NOS2^-^ tumor edges with an important distinction of low sporadic COX2 expression when compared to the type II immune desert. These types of tumor core low CD8^+^ T cell configurations offer descriptions of specific microcosms where tumor NOS2/COX2 distributions could have a regulatory role in the development of immune cold tumor regions. Mechanistically, COX2 inhibition by indomethacin augmented cytokines and chemokines that promote directional CD8^+^ T cell migration into the tumor core [2,31]. Further analysis of other biomarkers in these regions revealed NOS2^+^CD3^+^ lymphoid aggregates in type I immune deserts, as well as NOS2^+^CD31^+^αSMA^+^ vessels in type I and type II immune deserts [3]. Interestingly, iNOS-derived NO is known to induce T cell apoptosis and mediate vascular dysfunction [85,86]. Vascular formation under conditions of high iNOS leads to structural abnormalities including permeable, leaky vessels lacking proper integrity that augments nutrient delivery and tumor metastasis [19,87,88]. CD31 is important for lymphocyte trafficking and TLS formation, which predicts improved clinical outcomes in cancer [[89], [90], [91], [92], [93]]. Interestingly, Kashiwagi et al. have shown that silencing tumor nNOS led to normalized perivascular NO gradients and tumor vasculature, which improved tumor pO_2_ and radiation therapeutic efficacy [94]. Together, these observations offer additional insight into how elevated tumor NOS2 and COX2 can limit effective adaptive immunity within the tumor microenvironment, through reduced lymphocyte trafficking and increased lymphocyte apoptosis.

Transition from an “inflamed” CD8^+^ T-cell–rich state to an immune-desert or cold state is generally viewed as a progression toward a more aggressive, immune-evasive breast cancer microenvironment [3,5,23,28]. The spatial identification of immune deserts by MIF compares the tumor margin, extra-tumor space, and CD8^+^ T cell gradients into the tumor, which can be validated by pathologist-annotated H&E-stained serial sections [3]. The distribution profiles of CD8^+^, NOS2^+^, and COX2^+^ annotated regions can be spatially analyzed to generate density heat maps and distribution analyses that visualize CD8^+^ T cell gradients over the tumor periphery [3,5]. These strategies provide descriptions of CD8^+/−^NOS2^+/−^COX2^+/−^ and other phenotypes within the tumor immune microenvironment, which can identify specific cellular niches that contribute to immune suppression, cancer stemness and metastatic disease [3,5]. Importantly, S-UMAP analysis demonstrated the strong predictive power of tumor NOS2/COX2 clustering within a 25-50 μm radius in deceased patient tumors [5]. The identification of these immune desert phenotypes demonstrates a shift from high CD8^+^ T cell tumor infiltration in low NOS2/COX2 tumors and signifies a temporal progression from type I - III immune deserts during disease progression in NOS2/COX2 high tumors. The impact of these immune desert phenotypes relative to therapeutic response and survival have been reported [1,2,27]. Moreover, the expression patterns of extensive biomarker panels or libraries can be examined in this way to offer more profound descriptions of the tumor immune microenvironment (Fig. 5).

In contrast to immune deserts, fully inflamed phenotypes observed in NOS2/COX2 low tumors are further characterized by the presence of CD8^+^ T cells in both tumor and stroma regions, as well as proximal lymphoid aggregates or TLS [2,27,29]. These configurations exhibit increased levels of GRZMB and IFNγ, antigen presenting cells and TLS expressing CD8, CD4, CD20, and CD11c indicative of antitumor immune response [1,3,5].

**Type I immune desert (ID)** are characterized by > 50 μm gaps between the tumor margin and stroma restricted lymphoid aggregate structures (Fig. 5), where CD8^−^NOS2^+^COX2^+^ metastatic niches were identified [5]. These NOS2^+^COX2^+^ niches are associated with an increase in the elongation of tumor cells and their migration into lymphoid structures in the stroma, where it is plausible that NOS2-derived NO and COX2-derived PGE2 released from these NOS2^+^COX2^+^ tumor clusters could locally alter the immune polarization of neighboring immune cells as previously described [3,5,85,86]. These regions also exhibit increased EpCAM and CD44v6 stem cellular expression, along with ICAM1, which have been associated with poor survival [1,5]. While IFNγ is critical for tumor immune surveillance, the dysregulation of IFNγ signaling or the preferential expression of specific interferon stimulated genes (ISG) promotes immune evasion [95,96]. Importantly, the antitumor effects of INFγ depend on the extent of its spread, where overlapping IFNγ niches were associated with high numbers of CD8^+^ T cells [97]. Moreover, the induction of tumor NOS2 expression requires IFNγ and univariate analysis implicates IFNγ and tumor NOS2 as contributing factors for metastatic disease [4,5,59,79]. Given that NOS2 and COX2 perpetuate their expression through NOS2-derived NO and COX2-derived PGE2 feedforward signaling and PGE2 limits the directional migration of CD8^+^ T cells into the tumor core, type I immune desert regions could provide the perfect cocktail for metastatic disease progression [1,5,18,31].

Additional mechanisms associated with elevated NOS2-derived NO and COX2-derived PGE2 include increased IL10 and TGFβ immunosuppressive mediators that limit Th1 T cell secretion of IFNγ (Fig. 2) [20,61,98,99]. The dysregulation of IFNγ can also induce T_regs_ as well as cytolytic T cell exhaustion through autocrine PD-1 expression as well as IDO and PDL1 expressed on tumor and macrophages [3,85,86]. Spatial analysis implicated these factors in the disease progression of NOS2/COX2 high ER-breast tumors [3]. While low dispersed PD-L1 and IDO1 expression is observed in fully inflamed tumors associated with improved survival, their expression is more densely clustered in type I immune deserts [1,5].

**Type II and III immune deserts** are depleted of IFNγ producing lymphoid cells and are immunologically cold [3]. In addition, tumor NOS2 expression is abated in type II and III immune deserts due to the lack of infiltrating CD8^+^ T cells and IFNγ production [[3], [4], [5]]. Developing type II immune deserts express COX2 creating a barrier around the tumor core, which limits chemokine/cytokine expression required for directional migration of CD8^+^ T cell tumor infiltration [2,3,31]. When compared to control 4T1 tumor bearing mice, indomethacin or diclofenac NSAID treatment on a Nos2-background significantly augments the tumor infiltration of CD8^+^ T_eff_ cells that secrete IFNγ and GRZMB, which limits tumor growth and metastatic burden [6]. These results suggest that immune surveillance can be restored in immune cold tumors and that COX2 is a key factor inhibiting CD8^+^ T_eff_ cell penetration into the tumor [6].

In contrast to the developing type II regions, type III mature immune deserts exhibit abated COX2 expression with elevated levels of B7H4 (Fig. 5), which also impairs cytolytic CD8^+^ T cell function and borders necrotic and hypoxic tumor regions [1,3,100]. Limited COX2 expression in these regions is often associated with a paucity of cytokine producing immune cells. Given that hypoxia-induced B7H4 expression borders necrotic/hypoxic tumor regions [101] while COX2 requires oxygen to generate PGE2, the transition from COX2 to B7H4 tumor expression in hypoxic regions could provide an important mechanism for the maintenance of these immune cold tumor regions.

Immune desert regions are linked with elevated cholesterol biosynthesis, which negatively regulates type I interferons required for improved therapeutic efficacy, as well as TGFβ-rich fibrotic signatures [1]. Further examination of type III immune deserts reveals a progressive increase in tumor nodules encased in αSMA, a cancer-associated fibroblast marker as well as elevated Keratin14 tumor expression (Fig. 5) that have been linked with TGFβ signaling [102,103]. Moreover, Duan et al. have developed a TGF-β signaling-related lncRNA signature for predicting immune microenvironments that maintain immune cold regions that could be useful for the identification of TGF-β-associated targets [104]. A significant body of research has investigated the role of TGFβ in breast cancer [105,106], highlighting its association with various mechanisms of cancer stemness and metastasis, as well as its ability to induce COX2. Interestingly, TGFβ was identified as a dual regulator of COX2/PGE2 and tumor promotion in colorectal cancer cells in a RAS/Wnt/βCatenin-dependent manner [107]. Together, these results suggest a regulatory role of hypoxia and COX2/B7H4/TGFβ in the maintenance of immune deserts in these tumors.

The results of cell culture experiments summarized in sections 2, 3 above could further shed light on the dynamics involved during the development and maintenance of immune desert regions. Type I immune deserts associated with stroma restricted CD8^+^ T cells provide a source of IFNγ and TNFα that induces both NOS2 and COX2 tumor cell expression, which can further support one another through feedforward signaling [4,18]. However, due to abated CD8^+^ T cell infiltration, type II and III immune deserts lack both IFNγ and NOS2. While it is not clear why NOS2/COX2 feedforward signaling does not induce NOS2 in type II and III immune deserts that express COX2, it is plausible that the decline of COX2 expression as well as reduced oxygen gradient during transition from type II to type III does not provide enough COX2-derived PGE2 for NOS2 induction by PGE2 in these regions. Therefore, in this tumor immune microenvironment, B7H4 expression that is induced by hypoxia [101] and observed in type II and III immune deserts [3] takes over.

**Progression of Type I-III immune desert is a result of timing and spatial distribution of IFNγ/Th1 cytokines.** The progression from type I through type III is a working hypothesis that provides a signature of chronic inflammation in the tumor microenvironment from IFNγ Th1 stimulation to immunosuppression. This inflammatory progression of immune deserts can also be impacted by oncogenic and metabolic stressors that increase metastatic potential and therapy resistance [108]. Transient inflammation facilitates dysregulated cycles of IFNγ/TNF, which expand and fortify the depth of these immune deserts.

These spatial immune deserts mirror progression of classical molecular pathways in cascades of cells *in vitro* and other pathophysiological conditions induced by Th1 cytokines. IFNγ/Th1 cytokines induce NOS2 and COX2 expression in macrophages, fibroblasts, epithelial cells, or tumors with different temporal responses. In mouse macrophages, this induction occurs within 4-8 h; however, in human tumor cells it takes 24-48 h [4,59], which suggests different temporal NOS2 and COX2 roles in different cellular beds. Both NO and PGE2 have a concentration-dependent impact on the immune system. cGMP-dependent NO concentrations <50 nM [48] or <100 nM PGE2 augments the number and maturation of Th1 CD4^+^ and CD8^+^ T cells. In contrast, cGMP-independent NO > 100 nM and > 1 μM PGE2 leads to immune suppression with increased IL10 and TGFβ [17]. In the tumor bed, these higher gradients arise from increased clustering of NOS2- and COX2-expressing tumor cells yielding higher regional NO and PGE2 gradients [3]. In contrast, COX2^+^ macrophages are dispersed in lymphoid aggregates suggesting a lower flux of PGE2, which can increase IFNγ production from CD4^+^ and CD8^+^ and activate B cells compared to higher PGE2 from COX2^+^ tumor clusters, which are immunosuppressive [48,109].

**Role of P53**. Elevated NOS2 expression and NOS2-derived NO stabilizes p53 through activation of ATM and ATR [[110], [111], [112]]. Thus, tumor NOS2 induction and subsequent p53 stabilization promotes apoptosis in p53 competent tumor cells. In addition, p53 also regulates innate and adaptive immunity in the tumor microenvironment [65,[113], [114], [115]]. The stabilization of p53 downregulates NOS2 and COX2 expression returning cells to a non-inflamed state. However, p53 mutation, which can be induced by NOS2-derived NO [[116], [117], [118], [119]], hinders NOS2 and COX2 downregulation resulting in the sustained expression of NOS2 and COX2 in tumors and promotes immune evasion through abated recruitment of immune cells to the tumor site [65]. Meanwhile, macrophages and other host cells that retain functional p53 maintain cell death signaling. Apoptotic death of M1 and other proinflammatory immune cells along with increased TGFβ and IL10 prevents further induction of M1 responses, thus suppressing antitumor immunity (Fig. 2, Fig. 3). Consequently, NOS2 and COX2 levels in the tumor parenchyma remain elevated and persistent, exacerbating the formation of immune deserts. Thus, NOS2 and COX2 expression is concurrent with the cytokine flux.

## 7 The implications of tumor NOS2 and COX2 for advanced cancer treatment

The goal of immune therapy is to augment infiltration of cytolytic T_eff_ cells into the tumor core. A promising clinical trial employed the pan NOS inhibitor L-NMMA combined with taxane and low dose aspirin to prevent thrombosis in chemoresistant TNBC [7]. The overall response rate in all patients was approximately 45.8%, and 81.8% in patients with locally advanced disease, with no grade ≥3 toxicities related to L-NMMA [7]. Moreover, plasma cytokine analysis implicated an anticancer response characterized by increased neutrophils, M1 macrophages, and B cells, as well as altered CD8^+^ T cells [7]. These observations are supported by an earlier study showing that NOS inhibition combined with single dose tumor irradiation augmented tumor growth that was mediated in part by the downregulation of IL-10 and augmented IFNγ, IL2, and IL12 [38]. Collectively, these findings indicate that pan NOS inhibitors significantly alter the immune landscape in both human and murine models favoring antitumor immunity.

Metaplastic breast cancer (MpBC) is a rare, highly aggressive, and chemoresistant disease with a dismal survival rate of eight months in patients with metastatic disease [42]. Studies have indicated that up to 41% of MpBC tumors harbor PI3K mutations, which can be targeted by the PI3K inhibitor Alpelisib. Given that NO activates numerous oncogenic signaling pathways including PI3K, the ongoing clinical trial (NCT05660083) incorporating L-NMMA with Alpelisib was strategically developed. While patient-derived MpBC cell lines and PDX models have demonstrated L-NMMA augments Alpelisib efficacy, in part by reversing epithelial-mesenchymal-transition (EMT) and decreasing cancer stemness, RNAseq/GSEA analysis also demonstrate decreased TGFβ signaling and augmented type I and type II interferons [42]. These results suggest that L-NMMA and Alpelisib combined therapy augment proinflammatory immune responses in treated tumors [42]. Interestingly, in addition to oncogenic pathway activation by NO, recent studies with the non-tumorigenic, P53 competent MCF10-A cells, have shown that NO induces mutations in PI3K (E545A), like the missense, gain-of-function PI3K mutation (E545K) identified in MpBC patients [42,116]. Both mutations lead to ligand-independent signaling, increased cell proliferation, glycolysis, which is immunosuppressive and induced by NO, and drug resistance.

## 8 Conclusions

The conversion of immune deserts in the 4T1 TNBC model to fully inflamed states by NOS2 and COX2 inhibition implicates these two proteins as cornerstones of tumor immune suppression, which is supported by past and ongoing clinical trials (NCT05660083) [2,6,7]. The 4T1 mouse model demonstrated that the NSAID indomethacin, which accumulates in tumors with high COX2 expression [6,120,121], can reduce tumor growth and metastatic burden [2,6]. Importantly, as high as 33% cure rate was observed in indomethacin-treated 4T1 tumor bearing Nos2-mice, where 40% of these mice resisted a second 4T1 tumor challenge [6]. Indomethacin significantly limited tumor growth and metastatic burden following surgical resection in Nos2-mice treated with docetaxel [6]. In addition, adjuvant COX inhibition by indomethacin significantly reduced tumor growth, lung metastasis and improved survival following single dose radiation of 4T1 tumor bearing mice, which involved at least in part augmented cGAS/STING signaling and augmented type I interferons [2]. Collectively, these studies show that dual treatment with NOS2 and COX2 inhibitors significantly enhances the adaptive immunity and immune memory with and without therapeutic treatment [2,3,[5], [6], [7]]. Therefore, NOS2 and COX2 inhibitors could offer clinically available alternatives for patients who have otherwise exhausted all treatment options.

## CRediT authorship contribution statement

**Lisa A. Ridnour:** Writing – review & editing. **Robert YS. Cheng:** Data curation. **William F. Heinz:** Formal analysis. **Leandro L. Coutinho:** Formal analysis. **Erika M. Palmieri:** Investigation. **M Cristina Rangel:** Resources. **Stephen K. Anderson:** Writing – review & editing. **Daniel W. McVicar:** Writing – review & editing. **Stephen M. Hewitt:** Writing – review & editing. **Stephen TC. Wong:** Software. **Xiaoxian Li:** Resources. **Stefan Ambs:** Resources. **Timothy R. Billiar:** Resources. **Sharon A. Glynn:** Formal analysis. **Jenny C. Chang:** Resources. **Stephen J. Lockett:** Resources, Writing – review & editing. **David A. Wink:** Conceptualization, Resources, Writing – original draft.

## Declaration of competing interest

X. Li reports personal fees from AstraZeneca, Roche, Eli Lilly and Company, and Onviv and grants from Champions Oncology outside the submitted work. T.R. Billiar reports a patent that describes the use of NOS2/COX2 inhibitors in liver cancer pending at the University of Pittsburgh. The other authors reported no disclosures. J. Chang is the sole inventor on patent application no. 10420838 entitled “Methods for treating cancer using iNOS-inhibitory compositions” held by Houston Methodist Hospital.

## Data Availability

Data will be made available on request.
